# Rapid Deployment of A Community Engagement Study And Educational Trial Via Social Media: Implementation of The UC-COVID Study

**DOI:** 10.21203/rs.3.rs-359099/v1

**Published:** 2021-05-07

**Authors:** Lauren E Wisk, Russell G. Buhr

**Affiliations:** David Geffen School of Medicine at the University of California

**Keywords:** coronavirus/COVID-19, Internet, social media, educational intervention, Crisis Standards of Care

## Abstract

**Background:**

In response to the COVID-19 pandemic and associated adoption of scarce resource allocation (SRA) policies, we sought to rapidly deploy a novel survey to ascertain community values and preferences for SRA, and to test the utility of a brief intervention to improve knowledge of and values alignment with a new SRA policy. Given social distancing and precipitous evolution of the pandemic, Internet enabled recruitment was deemed the best method to engage a community-based sample. We quantify the efficiency and acceptability of this Internet-based recruitment for engaging a trial cohort and describe the approach used for implementing a health-related trial entirely online using off-the-shelf tools.

**Methods:**

We recruited 1,971 adult participants (≥18 years) via engagement with community partners and organizations and outreach through direct and social media messaging. We quantified response rate and participant characteristics of our sample, examine sample representativeness, and evaluate potential non-response bias.

**Results:**

Recruitment was similarly derived from direct referral from partner organizations and broader social media based outreach, with extremely low study entry from organic (non-invited) search activity. Of social media platforms, Facebook was the highest yield recruitment source. Bot activity was present but minimal and identifiable through meta-data and engagement behavior. Recruited participants differed from broader populations in terms of sex, ethnicity, and education, but had similar prevalence of chronic conditions. Retention was satisfactory, with entrance into the first follow-up survey for 61% of those invited.

**Conclusions:**

We demonstrate that rapid recruitment into a longitudinal intervention trial via social media is feasible, efficient, and acceptable. Recruitment in conjunction with community partners representing target populations, and with outreach across multiple platforms, is recommended to optimize sample size and diversity. Trial implementation, engagement tracking, engagement and retention are feasible with off-the-shelf tools using preexisting platforms.

**Trial Registration:**

ClinicalTrials.gov registration NCT04373135.

## Background

The novel severe acute respiratory syndrome coronavirus (SARS-CoV-2) publicly emerged in December 2019 and has since rapidly spread throughout the world, constituting a major pandemic. Early in the pandemic, concern for health system capacity and virus containment prompted many health officials, universities, and hospitals to undertake development of scarce resource allocation (SRA) policies.(1) These policies outline rules for distribution of limited resources, such as ventilators or hospital beds, and aim to do so in a way that is both consistent and ethical, with imperatives for maximizing benefits, equal treatment, and prioritization on instrumental value and need that can be operationalized in a number of ways.(2) Though medical ethicists have written extensively on how to construct such a framework, there are limited studies that aim to evaluate and intervene on knowledge of and agreement with these ethical principles during an active pandemic when the threat of their application is extant.(3)

Conducting such a study requires reaching populations who may be the subject of allocation decisions and may be responsible for applying allocation decisions; both of which can be hard to reach during an active crisis. There is evidence that social media can be the best recruitment method for hard-to-reach populations and can be a similarly effective method as traditional recruitment in many cases.(4–7) Social media-delivered behavioral interventions have the potential to reduce the expense of more traditional interventions by eliminating the need for direct participant contact, also especially necessary during the pandemic, as well as increasing access to diverse participants that may not be accessible via clinic-based recruitment.(8) Although a burgeoning recruitment and delivery method, researchers are increasingly outlining rigorous methodological and ethical considerations for its use.(9–11) These methods have proven especially useful for the rapid recruitment into COVID-related studies during the current pandemic.(12)

We sought to utilize Internet-based methods for engaging community participants and health care workers in completing a trial testing an educational intervention designed to influence knowledge of SRA policies and trust in institutional implementation of these policies during an ongoing pandemic. In this report, we outline detailed methods for recruitment strategies and implementation of an entirely online trial, including considerations for data fidelity and ethical oversight, with the use of widely available tools; we further summarize strengths and limitations of our approach and make recommendations for utilization of these methods in future work.

## Methods

The UC-COVID (**U**nderstanding **C**ommunity **C**onsiderations, **O**pinions, **V**alues, **I**mpacts, and **D**ecisions for COVID-19) Study is a community engagement study undertaken to characterize health and access to care during the COVID-19 pandemic. This study features an educational trial component to test the ability of a novel intervention to impact knowledge of and trust in institutional capacity to implement ethical allocation of scarce resources. We adopted a broad social media based recruitment strategy where we collaborated with community organizations, disease advocacy groups, and professional societies to invite study participation by direct messaging from organizations to their members; we also employed targeted social media posts and referrals from participants. Though our recruitment strategy primarily focused on groups in California (71% of our sample), eligibility was not restricted by location and all adults (age ≥ 18) were eligible. Recruitment for the survey opened on 5/8/2020 and closed on 9/30/2020; though 99% of respondents entered the survey between May and August. This study was approved by the Institutional Review Board and informed consent was obtained from all participants. This study was registered with ClinicalTrials.gov (NCT04373135).

### Study Design and Procedures

To promote recruitment, we established partnerships with several disease advocacy groups (e.g., COPD Foundation, Taking Control of Your Diabetes, Pulmonary Hypertension Association, Vietnamese Cancer Foundation, AltaMed) and professional societies (e.g., California Thoracic Society, American Thoracic Society, Society for General Internal Medicine). Investigators contacted these groups, presented aligned goals, and proposed utilizing their networks for targeted study recruitment. Recruitment messages were primarily posted on social media accounts, message boards, and via direct newsletters to the email distribution lists of networks and partner groups. Broader study promotion was also achieved through ‘sharing’ of study information via personal/professional social networks of study investigators, colleagues/institutions, and participants (website analytics revealed that visitors shared study information 279 times via embedded share applet – similar to snowball sampling). Recruitment messages included IRB-approved language to promote the study (brief descriptions, inclusion criteria) and provide a link directing participants to a hosted study website. The study website included general study information (including IRB and trial registration information), research team contact information, and a share applet that allowed users to send an email or create a social media post linking back to the landing page. We tracked study website traffic (user and page views) during the recruitment period, including device on which the page was accessed (mobile, tablet, desktop), referral source, language, and location.

The study website directed participants to click an outbound link that transferred them to a Research Electronic Data Capture(13, 14) (REDCap, Nashville, TN; 5/8/20 to 6/19/20) or Qualtrics (Provo, UT) survey (6/19/20 to study close), hosted on secure servers at UCLA. Though we initially implemented our study in English only, to expand our ability to include diverse participants we utilized professional translation services (International Contact, Inc.; Berkeley, CA) to translate our study (website, consent, and survey) into the five most commonly spoken foreign languages in California (Spanish, Mandarin Chinese, Korean, Tagalog, and Vietnamese). As REDCap did not have native support for translation of the command buttons for the survey (e.g., “Next,” “Submit”), we migrated the survey to Qualtrics to facilitate full translation of the survey and interface.

**Figures 1:**
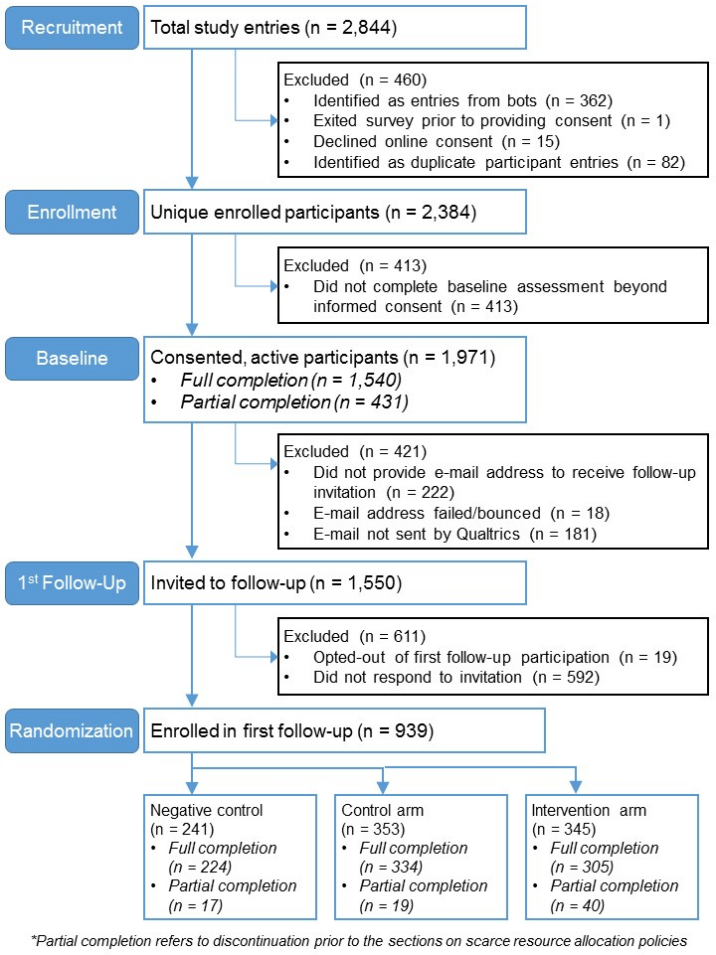
UC-COVID CONSORT Diagram CONSORT flow diagram showing participant flow from recruitment and into first follow-up and trial randomization.

After entering the survey, respondents first viewed an online consent form with language included in a typical written consent. Of the 2,844 survey initiations (Fig. 1), 362 (12.7%) entries from ‘bots’ and 82 (2.9%) duplicate participant entries were excluded; 2,384 respondents affirmed consent via the online form, 15 respondents declined consent, and 1 respondent exited the survey without affirming or declining consent. Of those who consented, 413 (17.3%) respondents did not continue the survey beyond that point, resulting in 1,971 (82.7%) consented, active participants. 1,540 (78.1%) participants completed the baseline assessment through at least part of the section on SRA policies, and thus were eligible for pre/post comparison of key trial outcomes.

Baseline participants who did not complete on their first attempt (and who provided an email address) received a reminder email four weeks after their last activity, then weekly for three weeks (for up to four total invitations) thereafter to remind/encourage survey completion.

### Assessing Data Validity

The Qualtrics platform has a built-in option (‘Prevent Ballot Box Stuffing’) that is designed to prevent duplicative entries by placing a cookie on the browser of participants during their first entry into the survey. If the same respondent comes back on the same browser and device, without having cleared their cookies, they are flagged as a duplicate and not permitted to take the survey again. However, clearing browser cookies, switching to a different web browser, using a different device, or using a browser in ‘incognito’ mode would all allow a participant to enter the survey again. As such, we additionally relied on embedded data to identify potential fraudulent entries for records attached to IP addresses that were duplicated in the data greater than four times; three of four instances were suspected to be the result of bots (fraudulent activity)(15) and discarded from the data. In the first instance, one IP address (geo-tagged to a location in China) contributed 172 attempted survey entries, none of which progressed in the survey beyond the consent page, that were all submitted within a 24-minute window. In the second and third instances, two IP addresses contributed 121 and 69 attempted survey entries, none of which progressed past the demographics section of the survey, all included similarly formatted email addresses (random word + four random letters @ domain), including some emails that were duplicated across these two IP addresses. The final instance included 19 records with unique and valid emails; these records were determined to be valid and submitted by unique individuals using a shared server. Invalid records submitted by bots were largely consistent with each other (e.g., 100% identified as health care workers, 100% reported their age reported an age between 30 and 33) and compared to valid records were more likely to report younger age, male sex, divorced/widowed/separated, having a bachelor’s degree, currently working, and having a military background (data not shown).

### Follow-Up Surveys

Following consent, respondents were asked to provide an email address for eligibility to receive a gift card and to receive follow-up surveys; respondents could still participate in the baseline survey if they did not provide an email (N = 222 did not provide an email). Follow-up invitations were sent in batches by month of baseline survey entry beginning in the second week of August so follow-up surveys were predominantly completed 2–3 months after the baseline survey; the first follow-up survey was closed in December. Participants received an email with a unique link to participate in the first follow-up survey; participants who did not complete the survey after the original invitation subsequently received a reminder email weekly for three additional attempts (for up to four total invitations) thereafter or until they completed. Of 1,749 provided e-mail addresses, only 1,550 invitations were initially sent as 18 emails were returned as undeliverable and by an unidentified error, 181 e-mails were marked as ‘not sent’ at the close of the pre-programmed Qualtrics distribution. Of follow-up invitations sent, 19 respondents opted-out/declined to participate in the follow-up survey and 592 did not respond to the follow-up requests, resulting in 939 (60.6%) entries into the first follow-up survey. Participants were sent a second follow-up survey via automated email invitation in January 2021, with up to 4 automated reminders to complete.

Participants who provided email addresses were entered in a raffle to win one of twenty-five (25) $100 gift cards for an online retailer; participants who complete two surveys receive one entry and those who complete all three surveys receive two entries.

### Intervention

During the first follow-up survey, respondents from California were automatically randomized to receive either a brief educational video explaining SRA policies or no intervention using a randomization module programmed into the survey that executed a stratified randomization scheme based on health care worker status, gender, age, race, ethnicity, and education. As the intervention was based on the policy developed by the University of California system (one of the largest providers in the state, with 10 campuses, five medical centers, and three affiliated national laboratories), participants outside California were treated as negative controls and not randomized.

Participants randomized to the intervention were automatically shown the intervention video, which was housed on a private Vimeo (New York, NY) channel and embedded in the survey. The 6:30 minute long video was animated by a professional video production company (WorldWise Productions, Los Angeles, CA) and covered key topic of public health ethics, policy development, and a summary of how the University of California’s SRA policy would be implemented during a crisis. A copy of the video is available upon request. In addition to viewing the intervention video, participants randomized to treatment were also shown five additional survey questions to assess their impressions of the intervention; all other content of the follow-up survey was identical to controls.

### Safety

At the completion of each survey, participants were directed to a “Thank you” page that additionally included a message directing them to contact the study team with any questions or concerns, including information on how to do so. Participants were also instructed to reach out to their personal health care or mental health provider if they experienced discomfort or distress, and provided with the website and phone number for the National Suicide Prevention Lifeline in the event they were in crisis.

### External Comparison

To determine the extent to which our sample is representative of the larger population from which our sample was drawn (primarily California adults, but also US adults), we compared our sample to respondents from 2019 Behavioral Risk Factor Surveillance System (BRFSS)(16). Our survey used a number of BRFSS questions (see below) to facilitate comparison.

### Survey Measures

Survey data included information on demographics (Sect. 1 & 6), health and health behaviors (Sect. 2), access to care (Sect. 3), experience with COVID-19/coronavirus (Sect. 4), and SRA policies (Sect. 5). The baselines questionnaire was the longest (approximately 35 minutes to complete) while subsequent surveys were designed to be shorter (approximately 15 minutes to complete).

Respondents first self-reported their status as a health care worker (*Are you a health care professional? Examples include: physician/doctor, nurse, pharmacist, respiratory therapist, rehab specialist*, *psychologist, clinical social worker, or hospital chaplain. If you are a health professional student* (*pre-degree or certificate*) *please select “no” for the purposes of this survey.*) Those who identified themselves as a health care worker received different survey items than non-health care workers. All participants were also asked to report their employment status, educational attainment, gender identity, year of birth, race, ethnicity, health insurance, place of residence, marital status, and if they had children. Health care workers were also asked to identify their specialty and tenure. A shorter version of this section was administered in the follow-up surveys to assess changes to employment and insurance.

The second section of the survey ascertained information on *health and health behaviors*; the majority of these questions were drawn from the BRFSS(16). Information included diagnosed chronic conditions, self-reported general health status (5-point Likert scale from ‘poor’ to ‘excellent’), number of days in the past 30 days where mental health was “not good” (“*Now thinking about your mental health, which includes stress, depression, and problems with emotions*…”) and where physical health was “not good” (“*Now thinking about your physical health, which includes physical illness and injury*…”), screeners for depression (PHQ-2)(17) and anxiety (GAD-2)(18), a single item on sleep from the PHQ-9(19), and were asked to compare their mental health now to the same time last year. Respondents were asked about alcohol use in the past 30 days (number of days of use, number of drinks per occasion), cigarette(20) and e-cigarette(21) use in the past 30 days, and exercise in the past 30 days; they were also asked to report if recent changes in these behaviors and if COVID-19 was a cause. A shorter version of this section was administered in the follow-up surveys to assess physical and mental health. Self-identified healthcare workers were also asked about burnout in subsequent surveys.(22)

The third section focused on access to care; all participants were asked about receipt of an influenza vaccination for the 2019 season, if they had a personal doctor, and the time they last saw their personal doctor. Those who previously reported any chronic medical condition were asked a set of novel questions about the impact of COVID-19 and related social distancing on their disease management and symptoms. Non-health care workers were asked an additional series of novel questions about changes in their ability to access health care during COVID-19, delayed or forgone care during COVID-19, and changes in the use of prescription and over-the-counter medications during COVID-19. This section was not administered during the first follow-up survey and an abbreviated version was administered at the second follow-up survey.

The fourth section focused on *COVID impacts* and asked respondents about their knowledge of government regulation of activities during the pandemic, personal protective behaviors, COVID-19 information seeking,(23) COVID-19 related stress,(23) and perceived personal risk from COVID-19. At follow-up, this section additionally contained questions about COVID testing and vaccination.

The fifth and longest section focused on awareness/knowledge of SRA policies, alignment with values governing SRA policies, preferences for SRA implementation and communication, and trust/anxiety for SRA. All SRA questions were novel but demonstrated acceptable psychometric properties (e.g., Cronbach’s alphas ranging from 0.5666 to 0.8954; Appendix 1). A shorter version of this section was administered in the follow-up surveys.

The final section asked optional personal questions regarding COVID-19 experiences(24) (exposure to COVID-19), disability status(25), advanced care planning, general sources of news/information(26), the experience of discrimination in health care, and other personal characteristics(27) (e.g., religion, sexual orientation, political identity). This section was not administered in the follow-up surveys.

### Statistical Analysis

All analyses were performed using SAS 9.4 (Cary, NC). Summary statistics were used to describe website traffic information based on analytics derived from the hosting platform (Square Space) and from Google analytics; correlation between website traffic and respondent counts by geography were evaluated. To ascertain the representativeness of the sample, we calculated and compared descriptive statistics for participants (N = 1,971), BRFSS respondents from California (N = 11,613), and BRFSS respondents from all 50 states and Washington DC (N = 409,810); BRFSS prevalence data are weighted to represent the US adult populations. Finally, differential non-responses was evaluated by comparing characteristics of those with complete (into SRA section) vs incomplete data at baseline. Differences were assessed using appropriate two-sided bivariate tests with a 0.05 alpha criterion.

## Results

**Figures 2:**
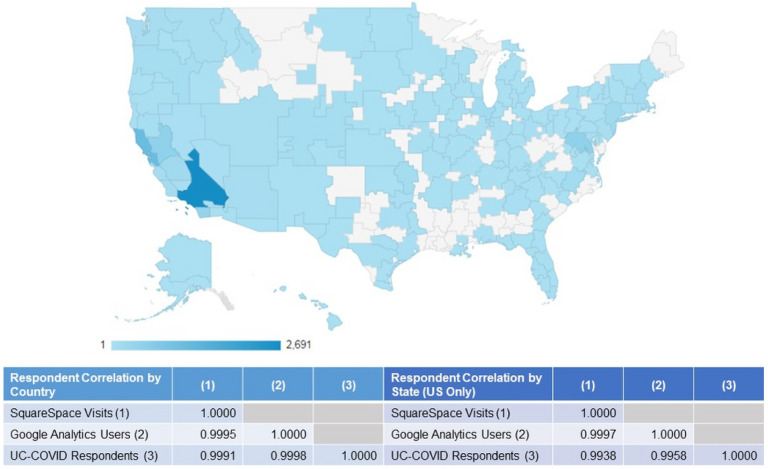
Geographic Coverage of UC-COVID Recruitment Google Analytics coverage map showing website traffic from 5/1/2020 to 9/30/30 from metro areas of the United States (traffic from outside the United States not shown) with inset table showing Pearson’s correlation between traffic from SquareSpace, traffic from Google Analytics, and respondents’ survey-reported residence at the country and state (US only) levels. Note: The designations employed and the presentation of the material on this map do not imply the expression of any opinion whatsoever on the part of Research Square concerning the legal status of any country, territory, city or area or of its authorities, or concerning the delimitation of its frontiers or boundaries. This map has been provided by the authors.

As about 1% of website traffic originated from organic searches, compared to the 55–60% from direct website entry or referral and 40–45% from social media recruitment, likelihood of un-invited survey participation (i.e., respondents who did not enter the survey as a direct result of targeted recruitment efforts) is relatively low (Table 1). Observed website traffic reflected the shift in recruitment strategy from outreach through partner organizations from May through July to broader social media based recruitment in August. Among social media platforms, Facebook was the highest yield recruitment source (85% of website traffic originating from social media). Website traffic was predominantly from California but still geographically diffuse, suggesting substantial national reach of recruitment efforts, and was highly correlated to place of residence reported by respondents, indicating similar response rates across geography (Fig. 2).

## Discussion

We demonstrate that rapid recruitment of a large cohort into a longitudinal educational trial via outreach through social media is feasible, efficient, and acceptable to participants. While recruitment through partner organizations was advantageous, broader social media based outreach was useful for expanding the number of recruited participants; diversified recruitment efforts may also be useful for maximizing sample diversity. Tracking of initial engagement with recruitment posts is facilitated by off-the-shelf software and preexisting platforms.

An emerging evidence base describes opportunities, such as rapidity, and shortcomings in Internet-enabled research, such as potential selection bias. This study is similarly subject to many of the potential biases reported elsewhere.(28, 29) Most notable are the potential issues with generalizability when the study sampling frame is an Internet-engaged audience;(30) though the digital divide has transformed over time,(31) there are clear selection biases stemming both from Internet-based recruitment and voluntary participation with limited remuneration. Indeed, we found that our sample was more likely to be female and less likely to be ethnically diverse, as with many Internet-recruited samples, but perhaps our biggest source of deviation from characteristics of the general adult population stemmed from targeted recruitment of health care workers,(32, 33) and thus over-representation of college-educated individuals. Examination of attrition revealed that loss from website engagement to survey participation was largely non-differential with respect to geography but that survey completion was linked to differences in many of the same characteristics that were associated with initial engagement. Despite these limitations, our sample was relatively representative with respect to age, certain racial subgroups, and importantly, presence of chronic medical conditions. Overall, such comparisons within and between the sample and target population, while not definitive, are helpful to assess the extent to which otherwise diffuse recruitment was effective for constructing a representative and minimally-biased sample (compared to expectations for Internet-recruited samples) and we strongly recommend designing similar comparisons in future work.

Despite these common sampling limitations with participatory research, we were still able to recruit and retain a number of respondents from smaller subgroups given our larger sample size and concerted efforts to partner with community organizations representing diverse subpopulations. Any future work in this area should strive to adopt a truly community-based participatory research (CBPR) approach in order to maximize reach among communities who are typically under-represented in research. We identified that offering to share data summaries with partners to “close the loop” and ensure shared use of study information, consistent with CBPR principles, was one effective method for engaging recruitment partners. Further efforts to improve recruitment and retention across socioeconomic strata could be enhanced by employing guaranteed remuneration.

In prior work using similar recruitment methods,(7, 15) identification of bots or other sources of invalid records is of particular concern and several methods for such identification have been utilized. We importantly identified that even with survey platform tools (such as the ‘Prevent Ballot Box Stuffing’ option), additional attention to records’ metadata is critical to identify residual entries submitted from bots. Future studies should consider how these tools could be applied in consort in order to generate a maximally valid sample.

## Conclusions

The unique challenges of diffuse recruitment via social media can raise concerns about efficiency, generalizability, and validity. We demonstrate the feasibility of implementing a rapidly deployed yet rigorous trial that engaged and retained a large cohort using off-the-shelf tools. In light of these successes in virtually recruiting for and conducting an educational trial, researchers may wish to implement similar strategies and reporting methods for future studies.

## Supplementary Material

Supplement 1

Supplement 2

## Figures and Tables

**Table 1 –:** Recruitment Tracking via Website Analytics

	Total^a^	May	June	July	August	September
**Google Analytics**						
**Users**^b^	8,069	2,767	550	963	3,427	541
**Sessions**^c^	10,439	3,549	761	1,254	4,312	563
**Sessions per User**	1.29	1.28	1.38	1.30	1.26	1.04
**Pageviews**^d^	15,769	4,081	1,659	1,966	7,157	906
**Pages per Session**	1.51	1.15	2.18	1.57	1.66	1.61
**User Acquisition Channel**						
Social	3,692 (45%)	1,230 (44%)	125 (23%)	228 (24%)	2,096 (62%)	13 (02%)
Direct	2,946 (36%)	1,290 (47%)	343 (64%)	512 (54%)	737 (22%)	64 (12%)
Referral	1,320 (16%)	107 (04%)	38 (07%)	188 (20%)	542 (16%)	445 (85%)
Email	135 (02%)	129 (05%)	6 (01%)	-	-	-
Organic Search	60 (01%)	9 (< 1%)	22 (04%)	14 (01%)	13 (< 1%)	2 (< 1%)
**Users by Language**						
English	7,873 (96%)	2,746 (99%)	535 (97%)	872 (91%)	3,278 (96%)	534 (99%)
Other	295 (04%)	33 (01%)	15 (03%)	91 (09%)	149 (04%)	7 (01%)
**Users by Location**						
United States	7,429 (92%)	2,564 (93%)	508 (92%)	887 (92%)	3,385 (99%)	257 (48%)
California	5,134 (64%)	1,135 (41%)	290 (53%)	655 (68%)	2,981 (87%)	148 (27%)
Other	646 (08%)	204 (07%)	42 (08%)	76 (08%)	42 (01%)	284 (52%)
**Users by Device**						
Mobile	4,599 (56%)	1,336 (48%)	233 (42%)	464 (48%)	2,384 (70%)	205 (38%)
Desktop	3,061 (38%)	1,367 (49%)	309 (56%)	454 (47%)	682 (20%)	317 (59%)
Tablet	495 (06%)	65 (02%)	8 (01%)	45 (05%)	361 (11%)	19 (04%)
**Sessions by Social Source**						
Facebook	3,528 (84%)	975 (74%)	73 (50%)	161 (63%)	2,309 (94%)	10 (59%)
Twitter	516 (12%)	294 (22%)	64 (44%)	44 (17%)	113 (05%)	1 (06%)
LinkedIn	106 (03%)	46 (03%)	7 (05%)	41 (16%)	12 (< 1%)	-
Instagram	30 (01%)	-	3 (02%)	10 (04%)	11 (< 1%)	6 (35%)
**Times Study Shared**	279	107	67	13	86	6
**Square Space Analytics**						
**Visits**^e^	10,330	3,679	888	1,260	5,471	292
**Pageviews**^f^	15,350	4,237	1,515	2,061	9,262	336
**Visit Acquisition Channel**						
Social	4,024 (39%)	1,318 (36%)	146 (16%)	234 (19%)	2,313 (55%)	13 (04%)
Direct	4,157 (40%)	1,874 (51%)	608 (68%)	703 (56%)	876 (21%)	96 (33%)
Referral	2,081 (20%)	471 (13%)	106 (12%)	311 (25%)	1,013 (24%)	180 (62%)
Email	4 (< 1%)	4 (< 1%)	-	-	-	-
Organic Search	64 (01%)	12 (< 1%)	28 (03%)	12 (01%)	9 (< 1%)	3 (01%)
**Pageviews by Language**						
English	15,398 (99%)	4,313(100%)	1,543 (95%)	2,027 (98%)	7,181 (99%)	334 (98%)
Other	175 (01%)	-	79 (05%)	45 (02%)	43 (01%)	8 (02%)
**Visits by Location**						
United States	9,563 (93%)	3,263 (89%)	734 (83%)	1,135 (90%)	4,152 (99%)	279 (96%)
California	6,498 (63%)	1,531 (42%)	391 (44%)	837 (67%)	3,575 (85%)	164 (56%)
Other	763 (07%)	416 (11%)	153 (17%)	123 (10%)	58 (01%)	13 (04%)
**Visits by Device**						
Mobile	5,570 (54%)	1,667 (45%)	317 (36%)	581 (46%)	3,493 (64%)	93 (32%)
Desktop	4,207 (41%)	1,930 (52%)	564 (64%)	634 (50%)	1,525 (28%)	188 (64%)
Tablet	553 (05%)	82 (02%)	7 (01%)	45 (04%)	453 (08%)	11 (04%)
**Visits by Browser**						
Chrome	2,232 (22%)	951 (26%)	284 (32%)	340 (27%)	889 (16%)	108 (37%)
Other	8,094 (78%)	2,728 (74%)	603 (68%)	918 (73%)	4,579 (84%)	184 (63%)
**Visits by Social Source**						
Facebook	3,430 (85%)	974 (74%)	72 (49%)	156 (67%)	2,218 (96%)	10 (77%)
Twitter	459 (11%)	295 (22%)	66 (45%)	30 (13%)	68 (03%)	-
LinkedIn	110 (03%)	49 (04%)	5 (03%)	39 (17%)	17 (01%)	-
Instagram	25 (01%)	-	3 (02%)	9 (04%)	10 (< 1%)	3 (23%)

a. Total may not be the sum of individual months as single users could access the website repeatedly across time. Similarly, user data may not sum to column totals if the same user accessed the site via different entry points.

b. Google Analytics defines users as the number of unique identifiers (assigned via a unique, randomly generate string that gets stored in a browser cookie) who have initiated at least one session during a given time period. Using cookies allows analytics to identify unique users across browsing sessions, but it cannot identify unique users across different browsers or devices.

c. Google Analytics defines sessions as the period of time a user is actively engaged with the site.

d. Google Analytics defines pageviews as the total number of pages viewed. Repeated views of a single page are counted.

e. SquareSpace defines a visit as a single browsing session, and can encompass multiple page views. Visits are tracked with a browser cookie that expires after 30 minutes; as such, any hit from a single user within that 30-minute browsing session count as one visit and that one person can register multiple visits a day if they close their browser and return to the site at least 30 minutes later. For visitors using the “Do not track” Chrome browser option, every page they view is tracked as a new visitor so will inflate visit data.

f. SquareSpace defines a page view as how many actual page requests the site saw in a given time period. All full page loads count toward total page views, including views to separate pages within the site (e.g., study information page, survey exit/thank you page).

Characteristics of recruited participants differed from the population of California and from the US population as a whole (Table 2). Compared to the adult population of California, study participants (71.0% from California) were more likely to identify as female, less likely to identify as Hispanic, and more likely to report having a bachelor’s degree. Despite being slightly more likely to report health insurance coverage and having a personal health care provider, study participants did not differ from adults in California with respect to the prevalence of chronic conditions or the likelihood of having a routine checkup in the prior year. Similar distinctions were observed when comparing study participants to the adult population of the US.

**Table 2 –:** Comparison of UC-COVID and BRFSS Respondents: Assessment of Sample Representativeness

	UC-COVID	All CA (BRFSS)	All US (BRFSS)
**Total N (unweighted)**	1,971	11,613	409,810
**Age (years)**			
18–34	20.7%	20.8%	20.3%
35–49	31.1%	35.4%	32.9%
50–64	29.0%	24.6%	25.4%
65+	19.2%	19.2%	21.4%
**Sex**			
Male	24.3%	49.3%	48.7%
Female	75.7%	50.7%	51.3%
**Race/Ethnicity**			
Hispanic	11.8%	36.2%	16.6%
Black, non-Hispanic	4.3%	5.8%	12.1%
Asian/Pacific Islander, non-Hispanic	12.1%	16.2%	5.7%
AIAN/Other race, non-Hispanic	3.3%	2.6%	2.7%
White, non-Hispanic	68.5%	39.3%	63.0%
**Has Bachelor’s Degree or More**	83.8%	29.8%	28.5%
**Marital Status**			
Married or living with partner	69.8%	56.1%	55.5%
Divorced, widowed, or separated	12.9%	17.6%	19.9%
Never married	17.3%	26.4%	24.6%
**Has Children (< 18) in Household**	30.3%	38.8%	34.8%
**Is a Veteran or Active Duty Military**	4.8%	7.5%	10.2%
**Has Health Insurance Coverage**	93.5%	87.5%	87.0%
**Employment Status**			
Currently working	63.4%	59.0%	57.7%
Furloughed/On leave	6.7%	3.3%	2.8%
Retired or student	17.8%	23.0%	24.8%
Unable to work/Out of work	12.1%	14.7%	14.7%
**Has Chronic Condition**^a^	49.5%	48.4%	54.3%
**Has Personal Health Care Provider**	83.9%	74.8%	76.6%
**Had Routine Check-Up in Past Year**	73.8%	71.8%	76.6%
**Completed Survey in English**	98.9%	86.8%	93.8%

Study sample is compared to 2019 BRFSS respondents from California and from the whole Unites States (US). For BRFSs samples, survey weighted percentages are shown.

AIAN - American Indian/Alaska Native

a. Based on chronic conditions assessed in the BRFSS (ever diagnosis of): heart attack, heart disease, stroke, asthma, cancer, chronic obstructive pulmonary disease (COPD), arthritis, depression, kidney disease, and diabetes.

Respondents who did versus did not complete the baseline SRA questions also varied by sociodemographics (Table 3). Participants with complete SRA data were more likely to be younger, white non-Hispanic, have a bachelor’s degree, currently employed, married or partnered, insured, and living with a chronic condition.

**Table 3 –:** Comparison of UC-COVID Respondents by Baseline Completeness

	Baseline Complete?^a^		
	Yes	No	p-val
**Total N**	1,540	431	
**Health Care Worker**			0.018
No	69.0%	74.9%	
Yes	31.0%	25.1%	
**Age (years)**			0.003
18–34	21.2%	18.8%	
35–49	32.6%	25.8%	
50–64	28.3%	31.3%	
65+	17.9%	24.1%	
**Sex**			0.136
Male	25.1%	21.6%	
Female	74.9%	78.4%	
**Race/Ethnicity**			<0.001
Hispanic	9.9%	18.3%	
Black, non-Hispanic	4.3%	4.4%	
Asian/Pacific Islander, non-Hispanic	12.7%	9.7%	
AIAN/Other race, non-Hispanic	2.1%	7.7%	
White, non-Hispanic	71.0%	59.9%	
**Has Bachelor’s Degree or More**	86.1%	75.6%	<0.001
**Marital Status**			0.001
Married or living with partner	70.5%	67.3%	
Divorced, widowed, or separated	11.4%	18.1%	
Never married	18.1%	14.6%	
**Has Children (< 18) in Household**	30.9%	28.3%	0.299
**Is a Veteran or Active Duty Military**	5.0%	3.9%	0.363
**Has Health Insurance Coverage**	96.4%	83.3%	<0.001
**Employment Status**			<0.001
Currently working	65.7%	55.2%	
Furloughed/On leave	7.1%	5.3%	
Retired or student	16.1%	23.7%	
Unable to work/Out of work	11.1%	15.8%	
**Has Chronic Condition**^b^	51.4%	42.9%	0.002
**Has Personal Health Care Provider**	83.1%	89.0%	0.046
**Had Routine Check-Up in Past Year**	74.5%	69.5%	0.168
**Completed Survey in English**	98.9%	98.8%	0.922
**Place of Residence**			<0.001
California	68.5%	79.8%	
Other	31.5%	20.2%	
**Provided E-mail for Re-contact**	87.7%	92.3%	0.007

AIAN - American Indian/Alaska Native

a. Completeness refers to at least partial completeness for the scarce resource allocation policies questions

b. Ever diagnosis of: heart attack, heart disease, stroke, asthma, cancer, chronic obstructive pulmonary disease (COPD), arthritis, depression, kidney disease, and diabetes.

## References

[1] Cleveland Manchanda EC, Sanky C, Appel JM. Crisis Standards of Care in the USA: A Systematic Review and Implications for Equity Amidst COVID-19. J Racial Ethn Health Disparities. 2020. 10.1007/s40615-020-00840-5 PMC7425256 32789816

[2] Emanuel EJ, Persad G, Upshur R, Thome B, Parker M, Glickman A, Fair Allocation of Scarce Medical Resources in the Time of Covid-19. N Engl J Med. 2020;382(21):2049–55. 32202722 10.1056/NEJMsb2005114

[3] Fallucchi F, Faravelli M, Quercia S. Fair allocation of scarce medical resources in the time of COVID-19: what do people think? J Med Ethics. 2020. 10.1136/medethics-2020-106524 PMC7551740 33046588

[4] Topolovec-Vranic J, Natarajan K. The Use of Social Media in Recruitment for Medical Research Studies: A Scoping Review. J Med Internet Res. 2016;18(11):e286. 27821383 10.2196/jmir.5698 PMC5118584

[5] Khatri C, Chapman SJ, Glasbey J, Kelly M, Nepogodiev D, Bhangu A, Social media and internet driven study recruitment: evaluating a new model for promoting collaborator engagement and participation. PLoS One. 2015;10(3):e0118899. 25775005 10.1371/journal.pone.0118899 PMC4361707

[6] Darmawan I, Bakker C, Brockman TA, Patten CA, Eder M. The Role of Social Media in Enhancing Clinical Trial Recruitment: Scoping Review. J Med Internet Res. 2020;22(10):e22810. 33104015 10.2196/22810 PMC7652693

[7] Wisk LE, Nelson EB, Magane KM, Weitzman ER. Clinical Trial Recruitment and Retention of College Students with Type 1 Diabetes via Social Media: An Implementation Case Study. J Diabetes Sci Technol. 2019;13(3):445–56. 31010315 10.1177/1932296819839503 PMC6501540

[8] Pagoto S, Waring ME, May CN, Ding EY, Kunz WH, Hayes R, Adapting Behavioral Interventions for Social Media Delivery. J Med Internet Res. 2016;18(1):e24. 26825969 10.2196/jmir.5086 PMC4752690

[9] Gelinas L, Pierce R, Winkler S, Cohen IG, Lynch HF, Bierer BE. Using Social Media as a Research Recruitment Tool: Ethical Issues and Recommendations. Am J Bioeth. 2017;17(3):3–14. 10.1080/15265161.2016.1276644 PMC5324729 28207365

[10] Arigo D, Pagoto S, Carter-Harris L, Lillie SE, Nebeker C. Using social media for health research: Methodological and ethical considerations for recruitment and intervention delivery. Digit Health. 2018;4:2055207618771757. 29942634 10.1177/2055207618771757 PMC6016568

[11] Russomanno J, Patterson JG, Jabson Tree JM. Social Media Recruitment of Marginalized, Hard-to-Reach Populations: Development of Recruitment and Monitoring Guidelines. JMIR Public Health Surveill. 2019;5(4):e14886. 31789598 10.2196/14886 PMC6915454

[12] Ali SH, Foreman J, Capasso A, Jones AM, Tozan Y, DiClemente RJ. Social media as a recruitment platform for a nationwide online survey of COVID-19 knowledge, beliefs, and practices in the United States: methodology and feasibility analysis. BMC Med Res Methodol. 2020;20(1):116. 32404050 10.1186/s12874-020-01011-0 PMC7220591

[13] Harris PA, Taylor R, Thielke R, Payne J, Gonzalez N, Conde JG. Research electronic data capture (REDCap)—A metadata-driven methodology and workflow process for providing translational research informatics support. J Biomed Inform. 2009;42(2):377–81. 18929686 10.1016/j.jbi.2008.08.010 PMC2700030

[14] Harris PA, Taylor R, Minor BL, Elliott V, Fernandez M, O’Neal L, The REDCap consortium: Building an international community of software platform partners. J Biomed Inform. 2019;95:103208. 31078660 10.1016/j.jbi.2019.103208 PMC7254481

[15] Pozzar R, Hammer MJ, Underhill-Blazey M, Wright AA, Tulsky JA, Hong F, Threats of Bots and Other Bad Actors to Data Quality Following Research Participant Recruitment Through Social Media: Cross-Sectional Questionnaire. J Med Internet Res. 2020;22(10):e23021. 33026360 10.2196/23021 PMC7578815

[16] Pierannunzi C, Hu SS, Balluz L. A systematic review of publications assessing reliability and validity of the Behavioral Risk Factor Surveillance System (BRFSS), 2004–2011. BMC Med Res Methodol. 2013;13:49. 23522349 10.1186/1471-2288-13-49 PMC3622569

[17] Kroenke K, Spitzer RL, Williams JB. The Patient Health Questionnaire-2: validity of a two-item depression screener. Med Care. 2003;41(11):1284–92. 14583691 10.1097/01.MLR.0000093487.78664.3C

[18] Plummer F, Manea L, Trepel D, McMillan D. Screening for anxiety disorders with the GAD-7 and GAD-2: a systematic review and diagnostic metaanalysis. Gen Hosp Psychiatry. 2016;39:24–31. 26719105 10.1016/j.genhosppsych.2015.11.005

[19] Kroenke K, Spitzer RL, Williams JB. The PHQ-9: validity of a brief depression severity measure. J Gen Intern Med. 2001;16(9):606–13. 11556941 10.1046/j.1525-1497.2001.016009606.x PMC1495268

[20] Office on Smoking and Health. 2020 National Youth Tobacco Survey: Methodology Report. Atlanta, GA: U.S. Department of Health and Human Services, Centers for Disease Control and Prevention, National Center for Chronic Disease Prevention and Health Promotion, Office on Smoking and Health,; 2020.

[21] Pearson JL, Hitchman SC, Brose LS, Bauld L, Glasser AM, Villanti AC, Recommended core items to assess e-cigarette use in population-based surveys. Tob Control. 2018;27(3):341–6. 28624764 10.1136/tobaccocontrol-2016-053541 PMC6934252

[22] West CP, Dyrbye LN, Sloan JA, Shanafelt TD. Single item measures of emotional exhaustion and depersonalization are useful for assessing burnout in medical professionals. J Gen Intern Med. 2009;24(12):1318–21. 19802645 10.1007/s11606-009-1129-z PMC2787943

[23] Conway LGI, Woodard SR, Zubrod A. Social Psychological Measurements of COVID-19: Coronavirus Perceived Threat, Government Response, Impacts, and Experiences Questionnaires. PsyArXiv. 2020.

[24] Mehta S. COVID-19 Community Response Survey. Baltimore: Johns Hopkins University,; 2020.

[25] Brault MW. Review of Changes to the Measurement of Disability in the 2008 American Community Survey. United States Census Bureau; 2009 2009 September 22.

[26] Barthel M, Mitchell A, D. A-M, Kennedy C, Worden K. Measuring News Consumption in a Digital Era. Washington, D.C.: Pew Research Center; 2020 2020 December 8.

[27] California Health Interview Survey. CHIS 2020 Adult CAWI Questionnaire. Los Angeles, CA: UCLA Center for Health Policy Research; 2020 2020 October 20.

[28] Benedict C, Hahn AL, Diefenbach MA, Ford JS. Recruitment via social media: advantages and potential biases. Digit Health. 2019;5:2055207619867223. 31431827 10.1177/2055207619867223 PMC6685119

[29] Salvy SJ, Carandang K, Vigen CL, Concha-Chavez A, Sequeira PA, Blanchard J, Effectiveness of social media (Facebook), targeted mailing, and in-person solicitation for the recruitment of young adult in a diabetes self-management clinical trial. Clin Trials. 2020;17(6):664–74. 32627589 10.1177/1740774520933362 PMC7655669

[30] Chunara R, Wisk LE, Weitzman ER. Denominator Issues for Personally Generated Data in Population Health Monitoring. Am J Prev Med. 2017;52(4):549–53. 28012811 10.1016/j.amepre.2016.10.038 PMC5362284

[31] Gonzales A. The contemporary US digital divide: from initial access to technology maintenance. Information. Communication Society. 2016;19(2):234–48.

[32] Artiga S, Rae M, Pham O, Hamel L, Muñana C. COVID-19 Risks and Impacts Among Health Care Workers by Race/Ethnicity. Issue Brief. San Francisco, CA: Kaiser Family Foundation; 2020 November 11.

[33] Boniol M, McIsaac M, Xu L, Wuliji T, Diallo K, Campbell J. Gender equity in the health workforce: Analysis of 104 countries. Geneva: World Health Organization; 2019. /HWF/Gender/WP1/2019.1.WHO/HIS.

