# Supplementary material for: Rapid Deployment of A Community Engagement Study And Educational Trial Via Social Media: Implementation of The UC-COVID Study
Source: Res Sq. 2021 May 7:rs.3.rs-359099. Preprint. [Version 1] doi: 10.21203/rs.3.rs-359099/v1 (PMC8132248; doi:10.21203/rs.3.rs-359099/v1)
Supplement: Supplement 2 [file 2fc663daa3fbd866106eb0f2.docx]

**Appendix 1 – Psychometric Properties of Novel Scarce Resource Allocation Questions**

|  | **All** | **Lay** | **HCW** |
| --- | --- | --- | --- |
| **Values for Prioritization Logistics ^a^** | *0.6066* | *0.6068* | *0.6099* |
| They should try to save the most number of lives possible | 0.2787 | 0.2044 | 0.4528 |
| They should take life support away from some patients in order to give it to other patients who are more likely to survive | -0.0149 | -0.0564 | 0.0904 |
| They should make decisions on a first-come, first-served basis | 0.0898 | 0.1244 | 0.0051 |
| They should apply the same rules to decide who gets life support to all patients equally | 0.7052 | 0.7183 | 0.6810 |
| The same rules should apply to all patients even if they were admitted to the hospital before the crisis started | 0.8195 | 0.8356 | 0.7709 |
| The same rules should apply to all patients even if they're in the hospital for reasons that aren't related to the disaster or pandemic | 0.7888 | 0.8064 | 0.7483 |
| Hospital committees (instead of individual doctors) should make these decisions | 0.2057 | 0.2477 | 0.1213 |
| Hospital committees should not know the identities of the patients and use only medical information to make decisions | 0.3258 | 0.3129 | 0.3712 |
| Policies like this should be developed with input from patients and community members | 0.2393 | 0.2854 | 0.1379 |
| **Values for Prioritization on Health Factors ^b^** | *0.7698* | *0.7594* | *0.8523* |
| Patients who are deemed less likely to survive and make it out of the hospital alive | 0.6954 | 0.6777 | 0.7285 |
| Patients who have physical or intellectual disabilities | 0.5378 | 0.5096 | 0.5629 |
| Patients who have shorter expected lifespans because of chronic illness | 0.7769 | 0.7428 | 0.8268 |
| Patients who are elderly | 0.6978 | 0.6962 | 0.6795 |
| Patients who are children | -0.1289 | -0.1233 | -0.1597 |
| Patients expected to have a poor quality of life if they survive | 0.7230 | 0.7083 | 0.7906 |
| Patients expected to need life support for a long time to recover from their illness | 0.6569 | 0.7051 | 0.6198 |
| Patients who are chronically dependent on ventilators |  |  | 0.7013 |
| Patients in persistent vegetative or minimally conscious states |  |  | 0.7965 |
| **Values for Prioritization on Social Factors ^c^** | *0.8809* | *0.8720* | *0.8954* |
| People who are wealthy, famous, or in positions of power (for example: celebrities or politicians) | 0.2800 | 0.1813 | 0.4841 |
| People who are a racial or ethnic minority | 0.6435 | 0.6887 | 0.6315 |
| People who are LGBTQ+ (e.g, lesbian, gay, bisexual, or transgender) | 0.6333 | 0.6758 | 0.5865 |
| People who are prisoners | 0.6611 | 0.6590 | 0.6786 |
| People without health insurance | 0.6539 | 0.6511 | 0.6720 |
| People who are undocumented immigrants | 0.6978 | 0.7061 | 0.7012 |
| Patients who have shorter expected lifespans because of a serious health condition even if that condition is more common among people with a disability | 0.7862 | 0.7662 | 0.8025 |
| Patients who have shorter expected lifespans because of a serious health condition even if that condition is more common among racial or ethnic minorities | 0.8220 | 0.8397 | 0.7876 |
| Patients who have shorter expected lifespans because of a serious health condition even if that condition is more common among people living in poverty | 0.8114 | 0.8183 | 0.7923 |
| People who are philanthropic donors to the hospital or health system |  |  | 0.5067 |
| **Values for Prioritization Exemptions ^d^** | *0.8284* | *0.8243* | *0.8627* |
| Patients who are pregnant in the first trimester | 0.5288 | 0.5493 | 0.4904 |
| Patients who are pregnant in the third trimester | 0.5588 | 0.5889 | 0.4672 |
| First responders (for example: police, fire fighters) | 0.7526 | 0.7352 | 0.7557 |
| Health care workers in general who are critical to caring for patients | 0.8046 | 0.7981 | 0.7473 |
| Health care workers specifically who are on the front lines and at increased risk of harm from the pandemic | 0.7802 | 0.7964 | 0.6686 |
| Patients who are participating in medical research studies | 0.4851 | 0.4869 | 0.5153 |
| Patients who are the sole or only caregiver of a family member (for example: a child or a disabled or elderly relative) | 0.5866 | 0.5788 | 0.6019 |
| Members of the military or veterans | 0.6048 | 0.5858 | 0.6495 |
| Public officials (for example: a mayor, governor, president, or congressperson) | 0.3931 | 0.3359 | 0.5992 |
| Patients who are on the list to get an organ transplant | 0.1984 | 0.2436 | 0.2232 |
| Patients who recently received an organ transplant | 0.3876 | 0.4026 | 0.4441 |
| Patients who have recently undergone major surgery (not related to a transplant) |  |  | 0.5039 |
| Patients who have had a complication from medical care (for example: a procedural or surgical complication or adverse reaction to a medication) |  |  | 0.4420 |
| Families or friends of critical health workers |  |  | 0.4583 |
| **Preferences for Policy Disclosure ^e^** | *n/a* | *0.5795* | *0.6676* |
| Hospitals should make this information public so patients like me know what their policy is even if I never have to go to that hospital | 0.5655 | 0.5434 | 0.3472 |
| I would want a hospital to tell patients like me about their policy only if I were admitted and in critical condition | -0.2361 | -0.0380 | -0.0380 |
| I would consider policies like this when deciding if I would go to a certain hospital | 0.4666 | 0.5397 | 0.8641 |
| I would feel more at ease if my doctor verbally explained how a policy like this works |  | 0.6488 |  |
| I would feel more at ease if my doctor provided a written explanation of how a policy like this works |  | 0.6744 |  |
| I would consider policies like this when deciding where I would tell my friends or family to seek care |  |  | 0.9026 |
| I would consider policies like this when deciding where I would refer my patients for hospital care |  |  | 0.9020 |
| I would feel comfortable verbally explaining how a policy like this works to patients |  |  | 0.2156 |
| I would feel comfortable providing a written explanation of how a policy like this works to patients |  |  | 0.2615 |
| I would feel more comfortable if someone else other than me explained this policy to patients |  |  | 0.1395 |
| I would be comfortable explaining that a patient had to be taken off of a ventilator due to a policy decision to that patient or their family |  |  | 0.0647 |
| **Trust in Policy Implementation ^f^** | *0.5666* | *0.5671* | *0.7856* |
| I trust that hospitals and doctors will apply policies like this in a fair and consistent way | 0.8725 | 0.8883 | 0.7092 |
| I trust hospitals and doctors to be honest and transparent about how resources are used in a crisis | 0.8897 | 0.8998 | 0.7316 |
| I feel anxious or worried when I think about policies like these | -0.1574 | -0.1805 | -0.1966 |
| I would trust my doctors to be honest with me about my chances for survival if I were extremely ill or in critical condition | 0.5636 | 0.5854 | 0.7118 |
| I feel that I could be honest with a patient about their chances for survival |  |  | 0.5245 |
| I feel that my colleagues could be honest with a patient about their chances for survival |  |  | 0.6869 |
| I feel like I could apply policies like this in a fair and consistent way |  |  | 0.7258 |
| I feel like my colleagues could apply policies like this in a fair and consistent way |  |  | 0.8112 |
| I feel like my employer would support me if I had to make these types of decisions |  |  | 0.5690 |
| I feel like I would be distressed or uncomfortable if I had to carry out policies like these |  |  | -0.0840 |
| I feel like I would have adequate legal protection from fallout if I had to carry out policies like these |  |  | 0.3615 |
| I would have a moral objection to carrying out a policy like this |  |  | -0.1630 |

Standardized factor loadings for each item and Cronbach’s alphas for entire scales (in italics) are shown for the sample overall (‘All’ column), among a lay (non-HCW) sample (‘Lay’ column), and among a health care worker (HCW) sample (‘HCW’ column). Exploratory factor analysis revealed that single factor solutions were appropriate for most scales (Eigenvalues from 0.5933 [for ‘Preferences for Policy Disclosure’ among All respondents] to 4.5293 [for ‘Values for Prioritization on Social Factors’ among HCW] with a median of 2.8018).

1. Respondents were asked “*Please tell us how you feel hospitals and health care workers should make decisions like these in general.*” Items were rated from 1, I strongly disagree with this to 10, I strongly agree with this.
2. Respondents were asked “*How strongly do you feel the following health factors should influence how hospitals and health care workers decide who receives life support during a crisis like a disaster or pandemic?*” Items were rated from 1, Should be much less likely to get life support to 5, Should not influence one way or the other to 9, Should be much more likely to get life support.
3. Respondents were asked “*How strongly do you feel the following factors not related to a patient's health should be considered when making decisions about who should receive life support during a crisis like a disaster or pandemic?*” Items were rated from 1, Should be much less likely to get life support to 5, Should not influence one way or the other to 9, Should be much more likely to get life support.
4. Respondents were asked “*There may be some situations where exceptions are made for certain groups of people under policies like this. How strongly do you feel these groups should be considered when making decisions about who should receive life support during a crisis like a disaster or pandemic?*” Items were rated from 1, Should be much less likely to get life support to 5, Should not influence one way or the other to 9, Should be much more likely to get life support.
5. Respondents were asked “*How strongly do you agree or disagree with the following statements about how you would prefer to learn about such policies?*” Items were rated from 1, I strongly disagree with this to 10, I strongly agree with this. Given that there were only three items similarly available for both lay and HCW samples, overall Cronbach’s alpha is not reported.
6. Respondents were asked “*How strongly do you agree or disagree with the following statement about how policies like this would be applied?*” Items were rated from 1, I strongly disagree with this to 10, I strongly agree with this.
